# Alveolar Ridge Preservation Using Xenogeneic Collagen Matrix and Bone Allograft

**DOI:** 10.1155/2014/172854

**Published:** 2014-09-24

**Authors:** Andreas O. Parashis, Charalampos J. Kalaitzakis, Dimitris N. Tatakis, Konstantinos Tosios

**Affiliations:** ^1^Private Practice Limited to Periodontics, 33 Sp. Merkouri Street, 11634 Athens, Greece; ^2^Department of Periodontology, School of Dental Medicine, Tufts University, Boston, MA, USA; ^3^Division of Periodontology, College of Dentistry, The Ohio State University, Columbus, OH, USA; ^4^Department of Oral Pathology and Medicine, University of Athens, Athens, Greece

## Abstract

Alveolar ridge preservation (ARP) has been shown to prevent postextraction bone loss. The aim of this report is to highlight the clinical, radiographic, and histological outcomes following use of a bilayer xenogeneic collagen matrix (XCM) in combination with freeze-dried bone allograft (FDBA) for ARP. Nine patients were treated after extraction of 18 teeth. Following minimal flap elevation and atraumatic extraction, sockets were filled with FDBA. The XCM was adapted to cover the defect and 2-3 mm of adjacent bone and flaps were repositioned. Healing was uneventful in all cases, the XCM remained in place, and any matrix exposure was devoid of further complications. Exposed matrix portions were slowly vascularized and replaced by mature keratinized tissue within 2-3 months. Radiographic and clinical assessment indicated adequate volume of bone for implant placement, with all planned implants placed in acceptable positions. When fixed partial dentures were placed, restorations fulfilled aesthetic demands without requiring further augmentation procedures. Histological and immunohistochemical analysis from 9 sites (4 patients) indicated normal mucosa with complete incorporation of the matrix and absence of inflammatory response. The XCM + FDBA combination resulted in minimal complications and desirable soft and hard tissue therapeutic outcomes, suggesting the feasibility of this approach for ARP.

## 1. Introduction

Remodeling after tooth extraction results in substantial horizontal (3-4 mm) and vertical (1-2 mm) alveolar bone loss [1–3]. This postextraction alveolar bone loss can compromise or prevent subsequent implant placement, while loss of crestal support compromises the position and appearance of the soft tissues in aesthetic areas [2]. In an effort to minimize postextraction alveolar bone remodeling and thus prevent its undesirable sequelae, alveolar ridge preservation (ARP, a form of guided bone regeneration, GBR) was developed as a therapeutic modality.

Several studies have been conducted to evaluate the effectiveness of different ARP surgical techniques (e.g., flapped versus flapless and primary intention healing versus no primary closure) and materials (e.g., occlusive membranes and bone grafts), and several approaches have proven successful to varying degrees [1–16]. Systematic reviews indicate that the combination of bone grafts with resorbable membranes achieved the best results [1–3]. However, a high incidence of membrane exposure (potentially leading to infection), early membrane degradation with inadequate barrier function, postoperative discomfort with coronal flap movement, and loss of width and thickness of keratinized tissue in the alveolar ridge are among the reported complications [2, 6, 8, 17].

Soft tissue management is important for maintaining an appropriate soft tissue profile for future tooth- or implant-supported restorations [18–21]. For example, in patients with thin biotype, a connective tissue graft may be recommended during or after ARP [20]. In this context, a resorbable barrier that preserves soft tissue attributes could be a valuable addition to ARP protocols.

Recently, a new xenogeneic collagen matrix (XCM) (Mucograft, Geistlich Pharma AG, Wolhusen, Switzerland) of porcine origin has been introduced into clinical practice that is made of non-cross-linked collagen types I and III. The new XCM is designed with a bilayer structure to support tissue ingrowth, regeneration, and integration within the host tissue. It has been specifically designed for soft tissue regeneration and has been histologically and clinically evaluated for root coverage and keratinized tissue augmentation. Studies report complete integration and revascularization with mature mucosal and submucosal tissues after 3 months, in both nonsubmerged and submerged healing environments [22–33].

The structural characteristics of this XCM and the studies indicating that it maintains its barrier function for at least 30 days suggest that it could also be used as a GBR device [22, 23, 28]. Results of clinical studies suggesting that the width and thickness of keratinized tissue can be maintained or increased, even if this matrix is left exposed, indicate that this XCM may be used without coronal advancement of flaps or periosteal-releasing incisions [27, 29]. These are potentially significant advantages since such an approach could minimize the reported complications following GBR.

The aim of this case series is to report clinical, radiographic, and histological outcomes following use of a new XCM in combination with freeze-dried bone allograft (FDBA) for ARP.

## 2. Case Description and Results

### 2.1. Patient Characteristics

Nine nonsmoking patients (aged 21–74 years; 2 males and 7 females) were treated with ARP after extraction of 18 teeth in a private practice in Athens, Greece, from October 2010 to October 2012. Reasons for extraction were hopeless periodontal prognosis, endodontic failure, and nonrestorable tooth. All patients had a noncontributory medical history and required ARP either prior to future implant placement or for preservation of aesthetics prior to fabrication of a maxillary anterior tooth-supported fixed restoration (Table 1).

A presurgical evaluation, consisting of a detailed oral and periodontal examination and full mouth radiographic assessment, was conducted on all patients. Three patients presented with gingivitis, 4 presented with moderate or severe periodontitis, and 2 were on maintenance. All patients received at least one session of oral hygiene instruction and scaling prior to tooth extraction, in order to establish an oral environment more favorable to wound healing. In addition, periodontal treatment comprising of root planing under local anesthesia and access/regenerative surgery, when necessary, was completed prior to the ARP procedure. The recommended treatment was thoroughly explained to each patient and written informed consent was obtained.

### 2.2. Treatment Procedures and Histological Processing

**Figure 1:**
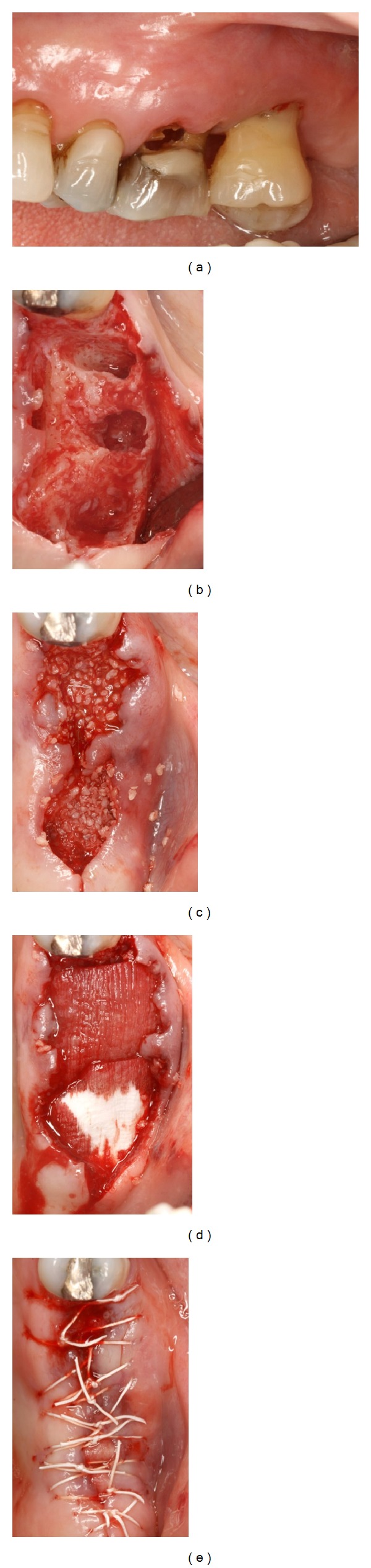
Clinical views of Case  1. (a) Pretreatment and after (b) extractions, (c) bone graft placement, (d) adaptation of two collagen matrices, and (e) suturing.

**Figure 2:**
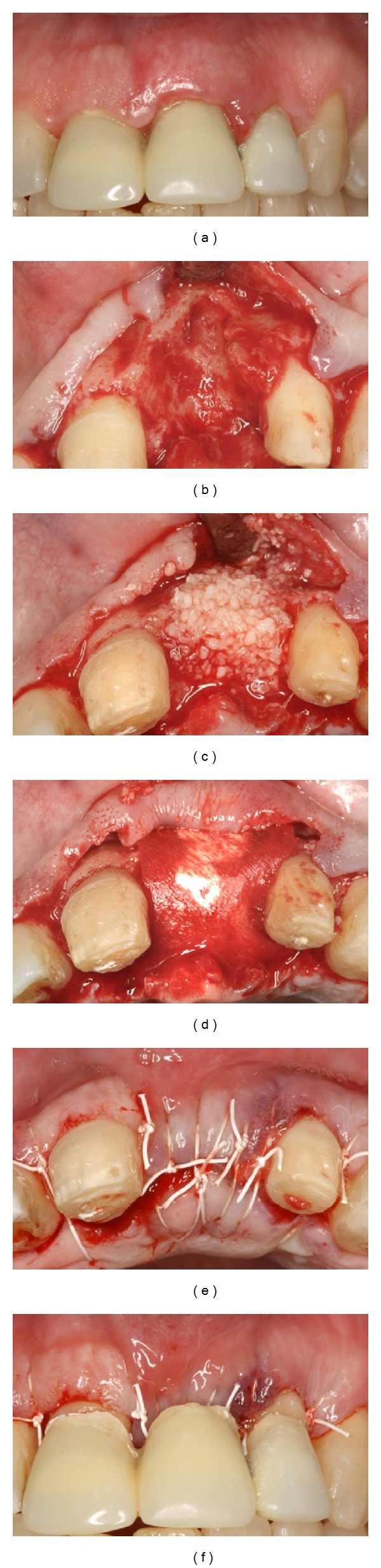
Clinical views of Case  2. (a) Pretreatment and after (b) extraction, (c) bone graft placement, (d) adaptation of the collagen matrix, (e) suturing, and (f) insertion of the temporary prosthesis.

**Figure 3:**
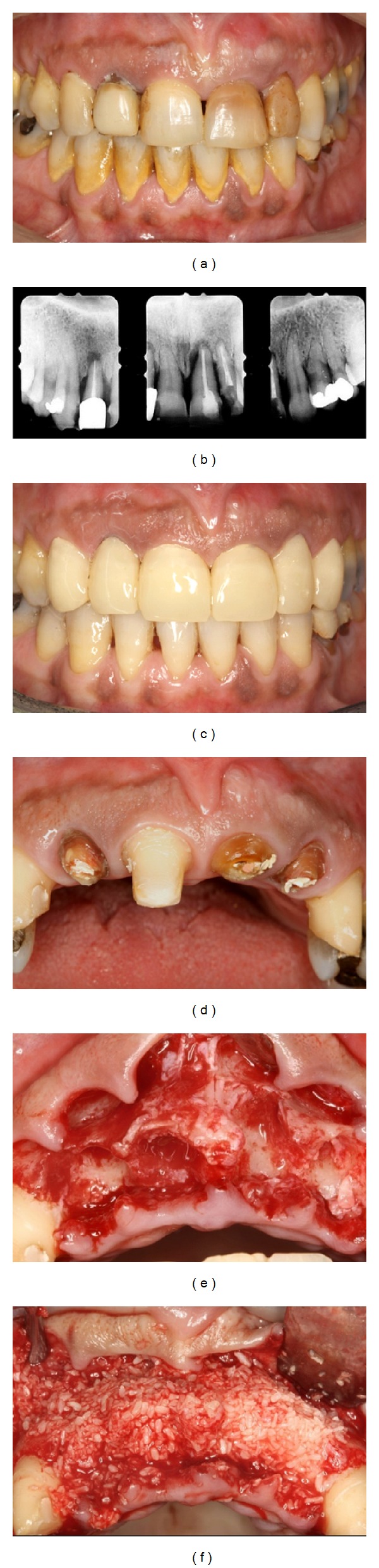
Case  3. Pretreatment: (a) clinical and (b) radiographic views. (c, d) Clinical views after periodontal treatment and construction of temporary prosthesis. After (e) extractions and (f) bone graft placement.

All patients received antibiotics (amoxicillin 500 mg three times a day for 8 days or azithromycin 250 mg once a day for 6 days) starting 2 days preoperatively. Following minimal flap elevation with soft tissue preservation, atraumatic extractions were performed, using piezosurgical instruments (Piezotome, Satelec Acteon, Merignac, France). Sockets were thoroughly degranulated and intramarrow penetrations were made. Buccal bone wall defects were present in all sockets, with a buccal bony dehiscence extending >50% of the length of the socket in seven sites. The FDBA (cortical bone (250–1000 *μ*m) LifeNet, Virginia Beach, VA, USA) was rehydrated and used to completely fill the sockets and resulting bony defects. The XCM was trimmed and adapted to cover the defects and 2-3 mm of the adjacent bone. Neither sutures nor fixation screws were used to stabilize the XCM. The flaps were repositioned (no periosteal release was performed) and sutured with nonabsorbable monofilament sutures (Gore-tex, W. L. Gore & Associates, Flagstaff, AZ, USA) (Figures 1, 2, 3, and 6). Postoperatively, patients were given instructions and prescriptions for antimicrobial rinse (chlorhexidine gluconate 0.12%) two times a day for 3 weeks and analgesics (acetaminophen 1000 mg or ibuprofen 400 mg) as needed for pain. Patients were seen at 1, 2, 4, 8, 12, and 24 weeks postoperatively. Sutures were removed at 2 weeks. Radiographic examination included periapical radiographs preoperatively and 6 months postoperatively and cone-beam computed tomography (CBCT) scans in 6 patients at 6 months.

Histological and immunohistochemical analysis included 9 sites from 4 patients 6 months after ARP (Table 1). Following thorough discussion and explanation of the procedure, patients agreed to proceed, and an informed consent form was signed that was based on the Helsinki Declaration of 1975, as revised in 2000. Soft tissues, approximately 3 × 4 mm in size, removed by soft tissue punches during guided implant placement surgery (Navigator System, Biomet 3i, Palm Beach Gardens, FL, USA) or ridge modification for construction of ovate pontics, were not discarded but placed in 10% buffered formalin, processed, and stored for future analysis. All specimens were analyzed after treatment of all patients was completed. After fixation in 10% buffered formalin, 5 *μ*m thick paraffin-embedded tissue sections were stained with hematoxylin and eosin. Streptavidin-biotin-peroxidase immunohistochemistry was performed with a fully automated slide preparation system (Ventana BenchMark XT, Ventana Medical Systems Inc., Tucson, AZ, USA) and commercially available detection kit (iView DAB, Ventana Medical Systems Inc., Tucson, AZ, USA). Antibodies used were Pan-keratin (dilution 1 : 50) (Clone AE1/AE3, Dako, Carpinteria, CA, USA) and CD34 (dilution 1 : 50) (Clone QBEnd 10, Dako, Carpinteria, CA, USA). Appropriate positive and negative (i.e., primary antibodies substituted with nonimmune serum) controls were used according to manufacturers' instructions.

### 2.3. Clinical and Radiographic Outcomes

**Figure 4:**
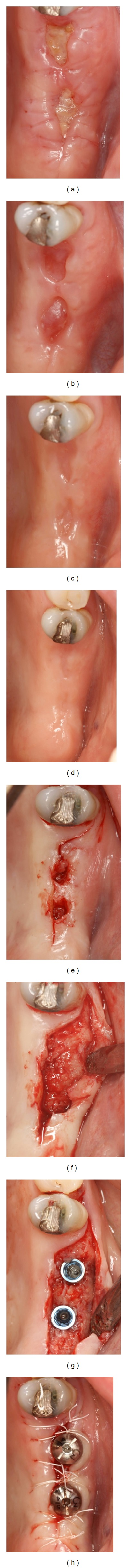
Clinical views of Case  1. Postoperative appearance at (a) 2 weeks, (b) 4 weeks, (c) 3 months, and (d) 6 months; after (e) soft tissue biopsies, (f) flap elevation, and (g, h) implant placement.

**Figure 5:**
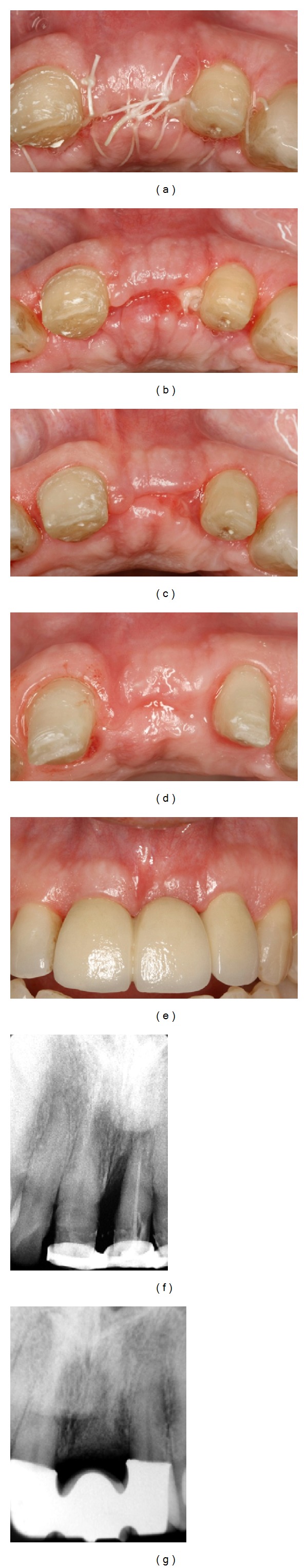
Clinical views of Case  2. Postoperative appearance at (a) 1 week, (b) 2 weeks, (c) 4 weeks, (d) 3 months, and (e) 6 months with the definitive prosthesis. Periapical radiographs (f) before extraction and (g) at 6 months postoperatively.

**Figure 6:**
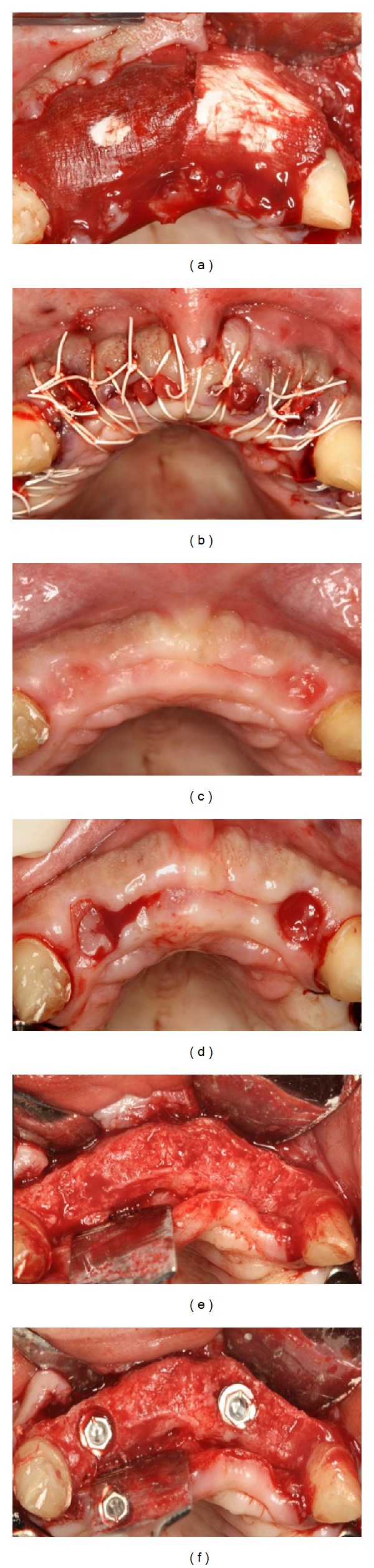
Clinical views of Case  3. After (a) adaptation of two collagen matrices and (b) suturing, (c) at 6 months, and after (d) soft tissue biopsies, (e) flap elevation, and (f) implant placement.

**Figure 7:**
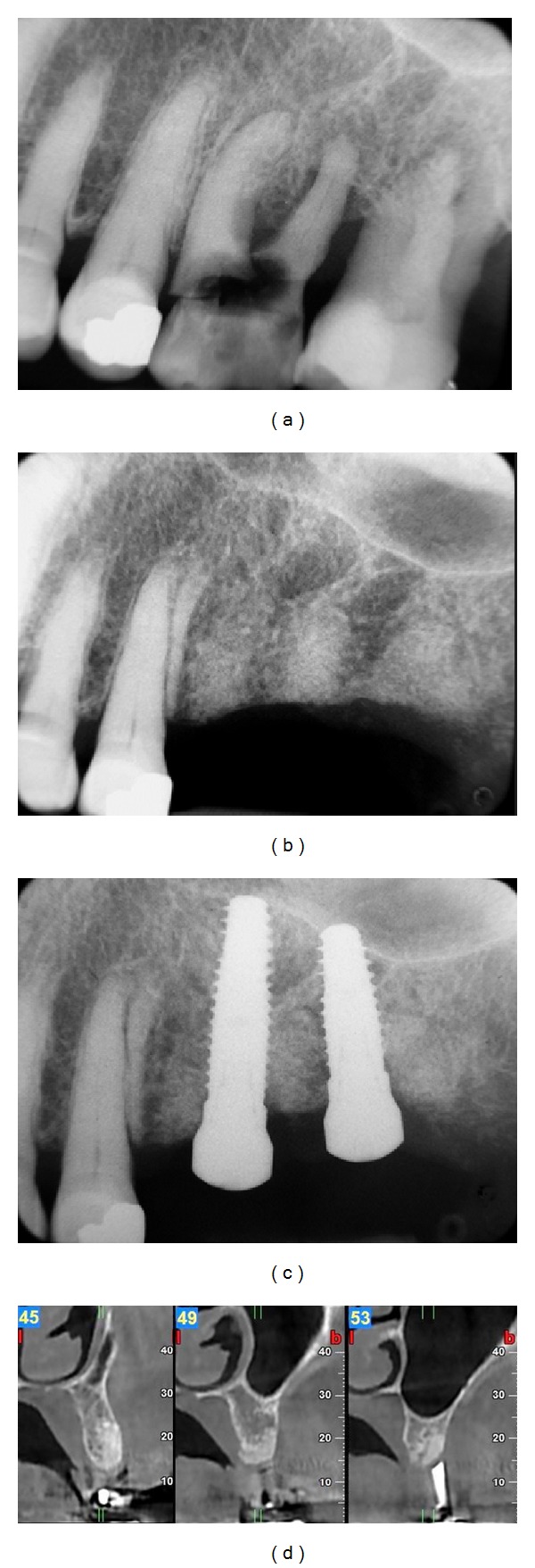
Radiographic views of Case  1. Periapical radiographs (a) before extractions, (b) at 6 months, and (c) after implant placement. (d) CBCT images at 6 months postoperatively.

**Figure 8:**
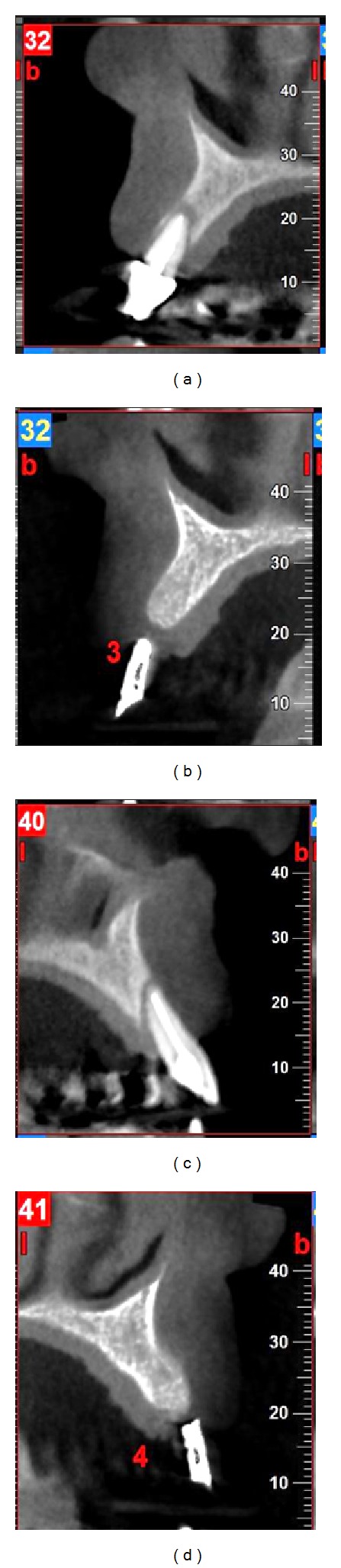
Radiographic views of Case  3. CBCT images at (a, c) pretreatment and (b, d) 6 months postoperatively.

All patients attended the scheduled postoperative visits and reported having followed the provided instructions. Postoperatively, patients reported only minor swelling and discomfort or pain; in all cases, healing was uneventful. Clinically, the XCM remained intact and any exposure was devoid of complications. The exposed matrix portions remained intact during the first 8 postoperative weeks and were slowly replaced by mature keratinized tissue within 2-3 months. The clinical appearance of the soft tissue between postoperative visits at 8 weeks and 24 weeks suggested that the width and thickness had increased (Figures 4 and 5). Radiographic assessment on CBCT scans at 24 weeks indicated adequate crestal bone width for standard-size implant placement in all sites, which was confirmed during surgery (Figures 4, 6, 7, and 8). Horizontal ridge width, measured with specialized software (SimPlant, Materialise Dental NV, Belgium) 1 mm below the most coronal aspect of the crest perpendicular to the long axis of the ridge, ranged from 5.8 to 7.6 mm. All treatment-planned implants were placed in acceptable positions and all fixed restorations fulfilled the aesthetic demands of the case without need for further soft or hard tissue augmentation procedures (Figure 5) (Table 1).

### 2.4. Histological Analysis

**Figure 9:**
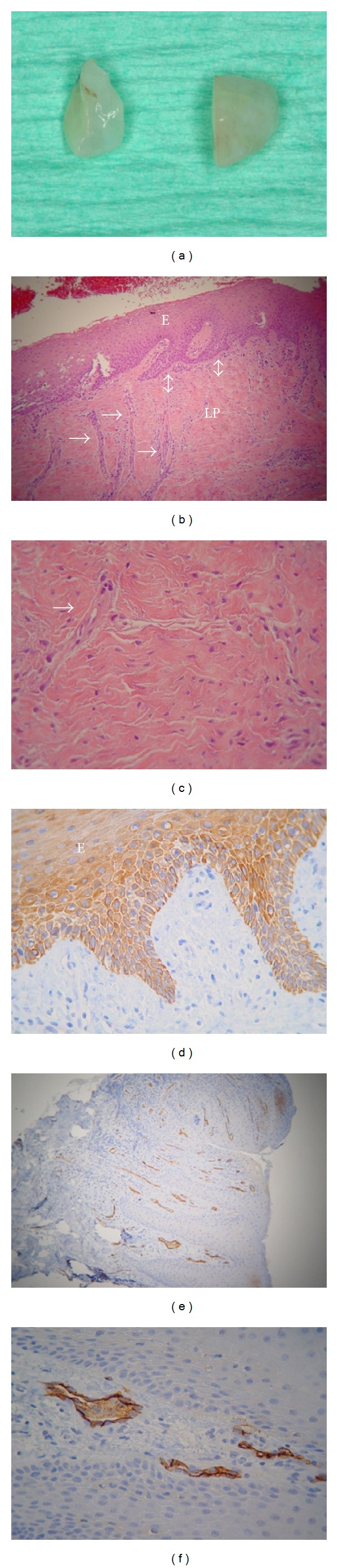
(a–c) Case  1, (d) Case  2, and (e, f) Case  3. (a) Gross tissue specimens. (b–f) Photomicrographs. (b) Normal-appearing oral mucosa, covered by parakeratinized squamous epithelium (E) with long interconnecting rete pegs (double arrows) and medium-sized vessels (arrows) (hematoxylin and eosin stain, original magnification ×200). (c) Lamina propria (LP) composed of fibrous connective tissue. Notice the many fibroblasts (purple nuclei) and a capillary vessel (arrow) (hematoxylin and eosin stain, original magnification ×400). (d) Pan-keratin-positive parakeratinized squamous epithelium (E) (streptavidin-biotin-peroxidase immunohistochemistry, original magnification ×200). (e, f) Normally distributed CD34-positive capillary vessels (brown) (streptavidin-biotin-peroxidase immunohistochemistry, original magnification (e) ×100, (f) ×400).

Following processing of the gross specimens (Figure 9(a)), microscopic examination showed that all specimens consisted of normal-appearing oral mucosa, covered by parakeratinized squamous epithelium with long interconnecting rete pegs (Figure 9(b)). The lamina propria was composed of fibrous connective tissue with interlacing bands of collagen fibers, showing many fibroblasts and medium- and small-sized vessels (Figure 9(c)). Neither foreign body reaction nor notable inflammatory infiltration was identified. The full thickness of the regenerated epithelium reacted with the Pan-keratin antibody (Figure 9(d)), while many capillary vessels lined by CD34-positive endothelial cells were regularly distributed in the lamina propria (Figures 9(e) and 9(f)).

## 3. Discussion

This case series reports on the clinical, radiographic, and histological outcomes after use of a new XCM and FDBA when performing ARP following tooth extraction. The treatment plan called for the treated sites to subsequently receive either dental implant placement or tooth-supported fixed partial dentures. The clinical and radiographic results indicated that the XCM-FDBA combination resulted in successful ARP outcomes, with minimal complications or patient discomfort and pain. The XCM remained clinically intact during the first 8 postoperative weeks; this suggests that the barrier function was maintained during the first 2 months of healing. Clinically, the exposed matrix portions were slowly replaced by mature keratinized tissue within 2-3 months, with tissue width and thickness seemingly increasing between the postoperative visits at 8 weeks and 24 weeks. Radiographic assessment at 24 weeks showed adequate crestal bone width for implant placement. All implants were placed in acceptable positions without need for further soft or hard tissue augmentation procedures, thus minimizing possible additional discomfort and cost for the patient during placement. The histological and immunohistochemical results indicated that the soft tissue regenerated over the extraction sites has the characteristics of normal keratinized oral mucosa.

These findings are consistent with some of the recent clinical [27, 29–31] and histological studies [24, 27] suggesting that the width and thickness of keratinized tissue can be maintained or even increased when this specific matrix is left exposed. The reported complete integration and formation of mature mucosal and submucosal tissues suggested that the new XCM could also be used as a GBR device [22, 23, 28] and that the XCM-FDBA combination could be an additional valuable option when performing ARP for subsequent implant placement or fixed partial denture restorative treatment. However, the limitations of this study—small and heterogeneous clinical, radiographic, and histological sample size; lack of clinical and preoperative radiographic measurements; and one time point of histological assessment—suggest caution in interpreting the results. Randomized controlled clinical trials are necessary to confirm the efficacy and predictability of this approach and to assess the long-term outcomes of implant therapy or conventional prosthodontics in sites treated with this protocol.

For a successful outcome in maintaining barrier function during the early postoperative healing period, resorbable barrier membranes used in GBR must possess certain properties, including biocompatibility, preferential tissue integration, place-holder characteristics, and adequate physicochemical stability. Collagen-based materials have often been explored for GBR applications because of the desirable material properties of collagen (natural origin, rapid biodegradation rate, biocompatibility, etc.). However, these same characteristics may prove to be a disadvantage, since barrier function may be limited over time [22]. To decrease the degradation rate and enhance the temporal stability of collagen-based membranes, manufacturers have used several cross-linking approaches [34]. Although cross-linking may address membrane stability in the oral or wound environment, it may also result in compromised attachment and proliferation of desirable connective tissue wound cells (e.g., fibroblasts), which could lead to delayed wound healing and possible infection [35] as well as to undesirable tissue reaction [36]. Therefore, alternative collagen processing and membrane manufacturing techniques have been developed. One such technique involves the combination of non-cross-linked native collagen III, which undergoes relatively fast degradation, and collagen I, which is more resistant, in order to tightly control membrane degradation [22]. In addition, the literature shows that intentional exposure of such bioresorbable membranes does not jeopardize the procedure outcomes (alveolar bone and keratinized tissue preservation) [2, 37].

The new non-cross-linked XCM is composed of collagen type I and type III without further cross-linking or chemical treatment. The XCM matrix is a bilayer: one side is thin and smooth and is of low porosity, while the other is a more porous 3-dimensional network. The XCM must be placed with the thin and smooth surface as the external layer since it is designed to allow cell attachment and host tissue integration but at the same time to remain impermeable to invading cells for 30 days. The more porous part is designed to be the internal layer since it is rapidly infiltrated by host mesenchymal cells [22, 23].

## 4. Conclusions

In summary, the favorable preliminary results reported here indicate that the XCM used could be a valuable alternative for ARP procedures. Randomized controlled clinical trials are necessary to confirm its efficacy and predictability and to assess the long-term outcomes of implant therapy or conventional prosthodontics in sites treated with this protocol.

## Figures and Tables

**Table 1:** Case demographics.

Case #	Age, sex	Tooth (#) extracted	Implant (#) placed	Tooth-supported restoration (#)	Biopsy site (#)
1	74, F	14, 15	14, 15	No	14, 15
2	50, F	9	No	8–10	9
3	52, F	4, 5, 7–10, 12, 13	4, 5, 7, 9, 12, 13	No	4, 5, 7, 10
4	66, F	3	3, 4	No	No
5	64, M	14	14, 15	No	No
6	65, F	9, 10	No	8–11	9, 10
7	29, M	8	No	6–11	No
8	36, F	8	8	No	No
9	21, F	6	6	No	No

## References

[1] Esposito M, Grusovin MG, Felice P, Karatzopoulos G, Worthington HV, Coulthard P (2009). Interventions for replacing missing teeth: horizontal and vertical bone augmentation techniques for dental implant treatment. *Cochrane Database of Systematic Reviews*.

[2] Heggeler JMT, Slot DE, van der Weijden GA (2011). Effect of socket preservation therapies following tooth extraction in non-molar regions in humans: a systematic review. *Clinical Oral Implants Research*.

[3] Hämmerle CHF, Araújo MG, Simion M (2012). Evidence-based knowledge on the biology and treatment of extraction sockets. *Clinical Oral Implants Research*.

[4] Lekovic V, Camargo PM, Klokkevold PR (1998). Preservation of alveolar bone in extraction sockets using bioabsorbable membranes. *Journal of Periodontology*.

[5] Artzi Z, Tal H, Dayan D (2000). Porous bovine bone mineral in healing of human extraction sockets. Part 1: histomorphometric evaluations at 9 months. *Journal of Periodontology*.

[6] Lasella JM, Greenwell H, Miller RL (2003). Ridge preservation with freeze-dried bone allograft and a collagen membrane compared to extraction alone for implant site development: a clinical and histologic study in humans. *Journal of Periodontology*.

[7] Griffin TJ, Cheung WS, Hirayama H (2004). Hard and soft tissue augmentation in implant therapy using acellular dermal matrix. *The International Journal of Periodontics & Restorative Dentistry*.

[8] Darby I, Chen S, De Poi R (2008). Ridge preservation: what is it and when should it be considered. *Australian Dental Journal*.

[9] Fickl S, Zuhr O, Wachtel H, Stappert CFJ, Stein JM, Hurzeler MB (2008). Dimensional changes of the alveolar ridge contour after different socket preservation techniques. *Journal of Clinical Periodontology*.

[10] Mardas N, Chadha V, Donos N (2010). Alveolar ridge preservation with guided bone regeneration and a synthetic bone substitute or a bovine-derived xenograft: a randomized, controlled clinical trial. *Clinical Oral Implants Research*.

[11] Weng D, Stock V, Schliephake H (2011). Are socket and ridge preservation techniques at the day of tooth extraction efficient in maintaining the tissues of the alveolar ridge?. *European Journal of Oral Implantology*.

[12] Brownfield LA, Weltman RL (2012). Ridge preservation with or without an osteoinductive allograft: a clinical, radiographic, micro-computed tomography, and histologic study evaluating dimensional changes and new bone formation of the alveolar ridge. *Journal of Periodontology*.

[13] Wood RA, Mealey BL (2012). Histologic comparison of healing after tooth extraction with ridge preservation using mineralized versus demineralized freeze-dried bone allograft. *Journal of Periodontology*.

[14] Leblebicioglu B, Salas M, Ort Y (2013). Determinants of alveolar ridge preservation differ by anatomic location. *Journal of Clinical Periodontology*.

[15] Cardaropoli D, Tamagnone L, Roffredo A, Gaveglio L, Cardaropoli G (2012). Socket preservation using bovine bone mineral and collagen membrane: a randomized controlled clinical trial with histologic analysis. * The International Journal of Periodontics & Restorative Dentistry*.

[16] Barone A, Toti P, Piattelli A, Iezzi G, Derchi G, Covani U (2014). Extraction socket healing in humans after ridge preservation techniques: comparison between flapless and flapped procedures in a randomized clinical trial. *Journal of Periodontology*.

[17] Lekovic V, Kenney EB, Weinlaender M (1997). A bone regenerative approach to alveolar ridge maintenance following tooth extraction. Report of 10 cases. *Journal of Periodontology*.

[18] Bouri A, Bissada N, Al-Zahrani MS, Faddoul F, Nouneh I (2008). Width of keratinized gingiva and the health status of the supporting tissues around dental implants. *The International Journal of Oral & Maxillofacial Implants*.

[19] Linkevicius T, Apse P, Grybauskas S, Puisys A (2009). The influence of soft tissue thickness on crestal bone changes around implants: a 1-year prospective controlled clinical trial. *The International Journal of Oral & Maxillofacial Implants*.

[20] Stimmelmayer M, Allen EP, Reichert TE, Iglhaut G (2010). Use of a combination epithelized-subepithelial connective tissue graft for closure and soft tissue augmentation of an extraction site following ridge preservation or implant placement: description of a technique. *The International Journal of Periodontics & Restorative Dentistry*.

[21] Fernandes PG, Novaes AB, de Queiroz AC (2011). Ridge preservation with acellular dermal matrix and anorganic bone matrix cell-binding peptide p-15 after tooth extraction in humans. *Journal of Periodontology*.

[22] Ghanaati S, Schlee M, Webber MJ (2011). Evaluation of the tissue reaction to a new bilayered collagen matrix *in vivo* and its translation to the clinic. *Biomedical Materials*.

[23] Jung RE, Hürzeler MB, Thoma DS, Khraisat A (2011). Local tolerance and efficiency of two prototype collagen matrices to increase the width of keratinized tissue. *Journal of Clinical Periodontology*.

[24] Camelo M, Nevins M, Nevins ML, Schupbach P, Kim DM (2012). Treatment of gingival recession defects with xenogenic collagen matrix: a histologic report. *The International Journal of Periodontics & Restorative Dentistry*.

[25] Rocchietta I, Schupbach P, Ghezzi C, Maschera E, Simion M (2012). Soft tissue integration of a porcine collagen membrane: an experimental study in pigs. *The International Journal of Periodontics & Restorative Dentistry*.

[26] Lorenzo R, Garcia V, Orsini M, Martin C, Sanz M (2012). Clinical efficacy of a xenogeneic collagen matrix in augmenting keratinized mucosa around implants: a randomized controlled prospective clinical trial. *Clinical Oral Implants Research*.

[27] Schmitt CM, Tudor C, Kiener K (2013). Vestibuloplasty: porcine collagen matrix versus free gingival graft: a clinical and histologic study. *Journal of Periodontology*.

[28] Cioban C, Zǎgǎnescu R, Roman A (2013). Early healing after ridge preservation with a new collagen matrix in dog extraction sockets: preliminary observations. *Romanian Journal of Morphology & Embryology*.

[29] Sanz M, Lorenzo R, Aranda JJ, Martin C, Orsini M (2009). Clinical evaluation of a new *collagen matrix* (Mucograft prototype) to enhance the width of keratinized tissue in patients with fixed prosthetic restorations: a randomized prospective clinical trial. *Journal of Clinical Periodontology*.

[30] McGuire MK, Scheyer ET (2010). Xenogeneic collagen matrix with coronally advanced flap compared to connective tissue with coronally advanced flap for the treatment of dehiscence-type recession defects. *Journal of Periodontology*.

[31] Thoma DS, Jung RE, Schneider D (2010). Soft tissue volume augmentation by the use of collagen-based matrices: a volumetric analysis. *Journal of Clinical Periodontology*.

[32] Jepsen K, Jepsen S, Zucchelli G (2013). Treatment of gingival recession defects with a coronally advanced flap and a xenogeneic collagen matrix: a multicenter randomized clinical trial. *Journal of Clinical Periodontology*.

[33] Cardaropoli D, Tamagnone L, Roffredo A, Gaveglio L (2012). Treatment of gingival recession defects using coronally advanced flap with a porcine collagen matrix compared to coronally advanced flap with connective tissue graft: a randomized controlled clinical trial. *Journal of Periodontology*.

[34] Goissis G, Marcantonio E, Marcantônio RAC, Lia RCC, Cancian DCJ, De Carvalho WM (1999). Biocompatibility studies of anionic collagen membranes with different degree of glutaraldehyde cross-linking. *Biomaterials*.

[35] Rothamel D, Schwarz F, Sculean A, Herten M, Scherbaum W, Becker J (2004). Biocompatibility of various collagen membranes in cultures of human PDL fibroblasts and human osteoblast-like cells. *Clinical Oral Implants Research*.

[36] Rothamel D, Schwarz F, Sager M, Herten M, Sculean A, Becker J (2005). Biodegradation of differently crosslinked collagen membranes: an experimental study in the rat. *Clinical Oral Implants Research*.

[37] Barone A, Ricci M, Tonelli P, Santini S, Covani U (2013). Tissue changes of extraction sockets in humans: a comparison of spontaneous healing vs. ridge preservation with secondary soft tissue healing. *Clinical Oral Implants Research*.

